# Comprehensive evaluation of cardiovascular risk control in a large national cohort: insights from over 1 million participants in a cardiovascular prevention program

**DOI:** 10.3389/fpubh.2026.1758928

**Published:** 2026-03-03

**Authors:** Grzegorz Kubielas, Izabella Uchmanowicz, Marcin Magdziarz, Michał Czapla, Katarzyna Kułaga, Piotr Dobrowolski, Aleksander Prejbisz

**Affiliations:** 1 Department of Nursing, Faculty of Nursing and Midwifery, Wroclaw Medical University, Wroclaw, Poland; 2 Department of Health Care Services, Polish National Health Fund, Central Office in Warsaw, Warsaw, Poland; 3 Centre for Cardiovascular Health, Edinburgh Napier University, Edinburgh, United Kingdom; 4 Hugo Steinhaus Center, Faculty of Pure and Applied Mathematics, Wrocław University of Science and Technology, Wrocław, Poland; 5 Department of Emergency Medical Service, Faculty of Nursing and Midwifery, Wroclaw Medical University, Wroclaw, Poland; 6 Group of Research in Care (GRUPAC), Faculty of Health Science, University of La Rioja, Logroño, Spain; 7 Nursing Care and Education Research Group (GRIECE), Nursing Department, University of Valencia, Valencia, Spain; 8 Department of Epidemiology, Cardiovascular Disease Prevention and Health Promotion, National Institute of Cardiology, Warszawa, Poland

**Keywords:** cardiovascular disease prevention, public health intervention, sex differences, smoking cessation, tobacco use disorder

## Abstract

**Introduction:**

Cardiovascular diseases (CVDs) remain the leading cause of mortality in Europe, driven by a persistently high prevalence of modifiable risk factors. The aim of this study is to asse**ss** the prevalence, age-related trends, and sex differences in cardiovascular risk factors among adults aged 35–65 years participating in the Cardiovascular Prevention Program in Poland (2022–2024).

**Methods:**

This cross-sectional study included 1,187,168 adults (718,528 women; 468,640 men) screened as part of Polish Cardiovascular Prevention Program. Evaluated risk factors were hypertension, hypercholesterolemia, elevated fasting glucose, tobacco use, low physical activity, overweight, and obesity. Analyses were stratified by age, sex, and year of participation. Linear and polynomial regression models were used to describe age-related patterns and short-term changes observed over the study period.

**Results:**

Overweight (46.9–47.0% vs. 33.3–34.0%; *p* < 0.001) and obesity (29.8–30.3% vs. 21.3–22.2%; *p* <0.001) were notably higher in men. Among women, overweight and obesity nearly doubled with age (*p* for trend < 0.05). Hypertension was less prevalent than expected but still higher in men (29.8–31.5% vs. 15.8–17.1%; *p* <0.001). Elevated cholesterol affected 63.5–66.9% of participants, especially women (*p* <0.001), with no age-related decline (*p* for trend < 0.05). Undiagnosed diabetes rose with age, reaching 5.1% in men aged 60–65 (*p* for trend < 0.001). Smoking persisted at high levels across all age groups, increasing among older women (*p* for trend < 0.05; sex difference *p* <0.001). Physical inactivity declined with age in women (*p* for trend < 0.05) but increased in men (*p* for trend < 0.05).

**Conclusion:**

In a large cohort without cardiovascular disease, we identified alarmingly high levels of modifiable risk factors, with significant age and sex disparities. These findings highlight the urgent need for targeted, sex- and age-specific strategies to reduce cardiovascular risk.

## 1 Introduction

Cardiovascular diseases (CVDs) remain the leading cause of mortality and morbidity worldwide, accounting for an estimated 17.9 million deaths annually and representing 32% of all global deaths (1). Across Europe, CVDs continue to place a substantial burden on healthcare systems, particularly in Central and Eastern European (CEE) countries, where premature mortality remains disproportionately high (2). In Poland, the burden of CVDs is particularly pronounced, with these conditions responsible for approximately 34.8% of all deaths in 2021 (around 160,000 annually), exceeding the European Union average of 31.4% (3, 4). This elevated cardiovascular mortality in Poland persists despite significant improvements in healthcare delivery and access to advanced treatments over the past three decades (5).

Recognizing these challenges, the Polish government has implemented several prevention initiatives. The Cardiovascular Prevention Program (pol. CHUK, Choroby Układu Sercowo Naczyniowego) represents one of the most comprehensive nationwide initiatives aimed at early detection and management of cardiovascular risk factors (6). The CHUK program, coordinated by the National Health Fund, is the largest nationwide primary prevention initiative in Poland, offering standardized cardiovascular risk assessment in asymptomatic adults aged 35–65 (6). Given the limitations in the existing literature, there is a clear need for comprehensive analyses based on large-scale, representative data. Despite insights from pan-European studies like EUROASPIRE V, population-based data on real-world primary prevention remain scarce, especially in CEE countries (7, 8).

This study aims to evaluate data from over 1 million adults enrolled in Poland’s CHUK prevention program (2022–2024) to assess the prevalence of major modifiable cardiovascular risk factors and unhealthy behaviors, exploring sex- and age-related patterns, and identifying emerging patterns that may inform more effective and targeted public health interventions. The findings are intended to support evidence-based decision-making for healthcare professionals and policymakers, enabling more efficient allocation of preventive resources toward high-risk populations in Poland. Beyond the national perspective, this study also contributes to European cardiovascular prevention strategies, in line with EU priorities to reduce disparities in non-communicable disease burden across member states.

## 2 Methods

### 2.1 Study design and data collection

This study presents a cross-sectional analysis of data collected through the CHUK prevention program in Poland between July 2022 and December 2024. It is a secondary analysis of routinely collected, anonymized administrative and clinical data derived from a nationwide cardiovascular prevention program implemented within the Polish public healthcare system. The program targets adults aged 35–65 years and provides cardiovascular risk assessment through standardized screening protocols implemented throughout the country. Screening assessments were conducted by primary healthcare providers delivering the program, and participation was voluntary following public information campaigns and invitations issued within primary care.

The analysis includes data from 1,187,168 participants who completed the screening during the study period. As noted in the methodology, the 2022 data include only the period from July 1 to December 31, 2022, while data for 2023 and 2024 cover the entire calendar years. Eligible participants for the CHUK program were men and women aged 35–65 years who had no prior diagnosis of type 1 or type 2 diabetes mellitus, chronic kidney disease (defined as eGFR <60 mL/min/1.73m^2^), familial hypercholesterolemia, or established cardiovascular disease, including coronary heart disease, cerebrovascular disease, or peripheral arterial disease. All individuals provided informed consent prior to enrolment. Participants were excluded if they were pregnant, required specialized cardiovascular care, or were unable to provide informed consent. Given the voluntary nature of the program and the evolving recruitment strategies over time, we analyzed data separately for each year (2022, 2023, and 2024). This approach accounts for potential differences in participant characteristics arising from temporal variation in enrollment dynamics. For example, individuals who enrolled early in the program may differ systematically from those recruited later through targeted outreach campaigns, including promotional materials and SMS invitations sent by primary care physicians. Accordingly, the CHUK program targets adults without previously diagnosed cardiovascular disease or diabetes. Therefore, the prevalence estimates reported in this study reflect screen-detected and previously unrecognized cardiovascular risk factors within a primary prevention cohort, rather than the overall population prevalence of these conditions.

### 2.2 Variables and measurements

The following cardiovascular risk factors and health behaviors were assessed as part of the study protocol implemented in the CHUK program. Hypertension was defined as a systolic blood pressure ≥140 mmHg and/or diastolic blood pressure ≥90 mmHg based on at least two independent measurements, or reported use of antihypertensive medication (9). All measurements were performed using certified and regularly inspected medical devices, in accordance with national regulations applicable to healthcare providers participating in the program. Elevated total cholesterol was defined as total cholesterol >190 mg/dL (10). Fasting glucose levels were classified as elevated when values exceeded 125 mg/dL. Smoking status was defined as current use of at least one cigarette per day, based on self-report. Physical activity was assessed via self-report and classified as low when individuals reported engaging in less than 150 min of moderate-intensity physical activity (e.g., walking, cycling, swimming) or less than 75 min of vigorous-intensity activity (e.g., running, aerobic classes) per week, or an equivalent combination of both. Overweight was defined as a body mass index (BMI) ranging from 25.0 to 29.9 kg/m^2^, while obesity was defined as a BMI equal to or greater than 30.0 kg/m^2^, in accordance with the World Health Organization (WHO) classification (11).

### 2.3 Ethical

The study was conducted in accordance with the Declaration of Helsinki. All procedures were carried out in compliance with the relevant guidelines and regulations. Ethical review and approval were not required in Poland for this study, as no identifiable data were collected and no medical interviews were conducted (12, 13). Consequently, approval from a bioethics committee was not necessary under Polish national legislation. STROBE (Strengthening the Reporting of Observational Studies in Epidemiology) guidelines were followed (14). Written informed consent for participation in a research study was not required, as the analysis was based on anonymized data collected as part of a routine, publicly funded health prevention program.

### 2.4 Statistical analysis

Statistical tests were conducted at a significance level of *α* = 0.05. Comparisons of proportions between participant groups were performed using chi-square tests. Analyses of variations across years were conducted using linear and non-linear regression models and Student’s t-tests. For age-related trend analyses, participants were stratified into six age groups: 35–39, 40–44, 45–49, 50–54, 55–59, and 60–65 years. Sex-specific analyses were performed for all variables to identify differential patterns between women and men. Linear regression models (*y* = *ax* + *b*) were fitted to assess trends where linear relationships were apparent, while polynomial regression models (*y* = *ax*^2^ + *bx* + c) were applied where non-linear patterns were observed. The coefficient of determination (2) was used to assess model fit, with values above 0.7 considered indicative of good fit. All analyses were performed using MATLAB R2024b and R 4.4.2 software packages on a Windows 10 Pro 64-bit system.

## 3 Results

### 3.1 Demographic characteristics

A total of 1,187,168 adults were screened in the CHUK program between 2022 and 2024, including 183,600 in the first half-year of implementation (July–December 2022), 572,551 in 2023, and 431,017 in 2024. Across all years, women predominated (59–63% of participants), with significant sex differences in enrolment confirmed by *χ*^2^ tests (*p* <0.001; Table 1).

**Table 1:** The number of participants in the CHUK program in the years 2022–2024.

The number of people surveyed						
Year	Total	Women		Men		*p*-value
	*n*	*n*	%	*n*	%	
2022^*^	183,600	115,868	63.11	67,732	36.89	<0.001
2023	572,551	347,743	60.74	224,808	39.26	<0.001
2024	431,017	254,917	59.14	176,100	40.86	<0.001

*Number of participants from July 1 to December 31, 2022.

### 3.2 Cardiovascular risk factors

Overweight and obesity were common across the study population, with clear sex differences. Nearly half of men were overweight (≈47%) compared with about one-third of women (≈33%), while obesity affected around 30% of men and 21% of women (*p* <0.001; Table 2).

**Table 2:** Trends in overweight and obesity by year and sex.

Overweight									
Year	Women				Men				*p*-value
	Yes		No		Yes		No		
	*n*	%	*n*	%	*n*	%	*n*	%	
2022^*^	39,334	33.95	76,534	66.05	31,797	46.95	35,935	53.05	<0.001
2023	116,866	33.61	230,877	66.39	105,392	46.88	119,416	53.12	<0.001
2024	84,936	33.32	169,981	66.68	82,533	46.87	93,567	53.13	<0.001

Obesity									
Year	Women				Men				*p*-value
	Yes		No		Yes		No		
	*n*	%	*n*	%	*n*	%	*n*	%	
2022*	25,219	21.77	90,649	78.23	20,200	29.82	47,532	70.18	<0.001
2023	77,082	22.17	270,661	77.83	68,033	30.26	156,775	69.74	<0.001
2024	54,365	21.33	200,552	78.67	52,419	29.77	123,681	70.23	<0.001

*Number of participants from July 1 to December 31, 2022.

**Figure 1:**
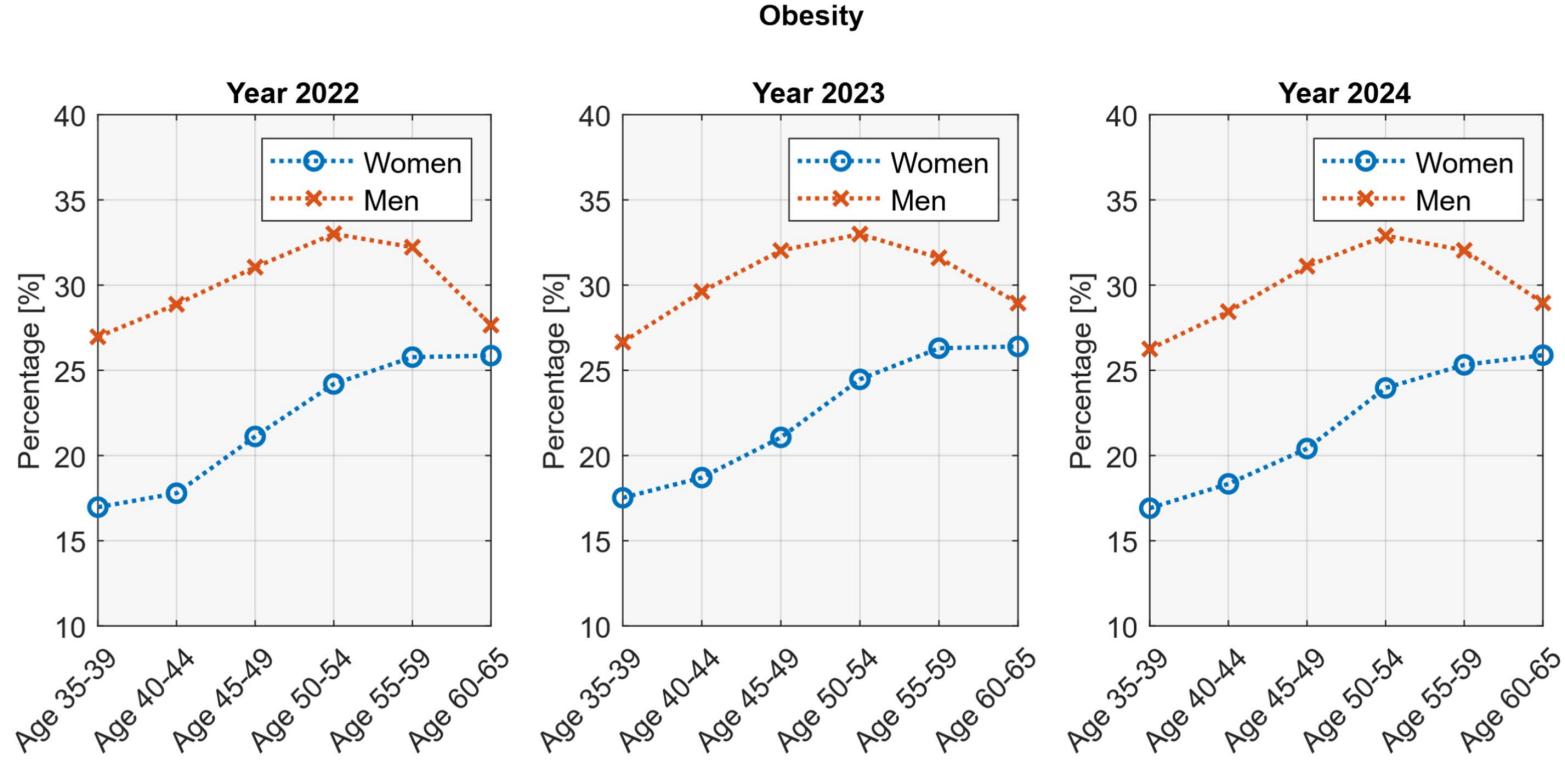
Age-stratified overweight and obesity rates by sex.

Age-stratified analyses revealed distinct sex-specific patterns. In women, overweight increased steadily from about one quarter at ages 35–39 to nearly 40% at ages 60–65 (*p* <0.05; *R*^2^ = 0.96–0.99). In men, overweight remained relatively stable across age groups. Obesity also rose progressively with age in women (*p* <0.05; *R*^2^ = 0.94–0.96), whereas in men it followed a non-linear trajectory, peaking at age 50–54 (≈33%) before declining in older groups (*p* <0.05; *R*^2^ = 0.86–0.99; Figure 1).

Hypertension affected around one fifth of participants overall (21–23%; Table 3). Men were consistently more likely to have hypertension than women, with prevalence close to one in three men (30–32%) compared with about one in six women (16–17%; *p* <0.001).

**Table 3:** Trends in hypertension prevalence.

Hypertension									
Year	Women				Men				*p*-value
	Yes		No		Yes		No		
	*n*	%	*n*	%	*n*	%	*n*	%	
2022*	19,593	16.91	96,275	83.09	20,738	30.62	46,994	69.38	<0.001
2023	59,445	17.09	288,298	82.91	70,787	31.49	154,021	68.51	<0.001
2024	40,207	15.77	214,710	84.23	52,439	29.78	123,661	70.22	<0.001

*Number of participants from July 1 to December 31, 2022.

**Figure 2:**
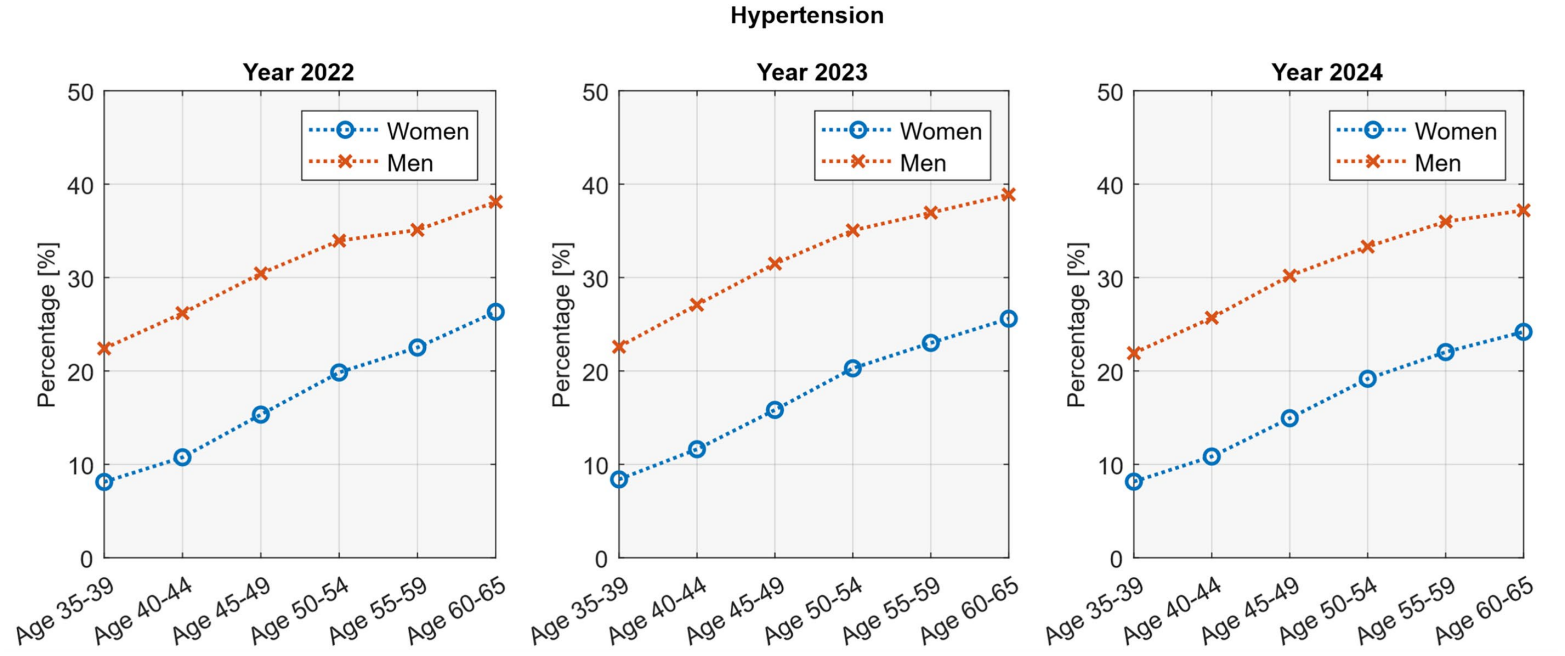
Age-stratified hypertension rates by sex.

Hypertension increased steadily with age in both sexes (Figure 2). In women, prevalence rose from about 8% at ages 35–39 to roughly one quarter at ages 60–65. In men, rates increased from just over one fifth at younger ages (22–23%) to nearly 40% at ages 60–65. Linear regression confirmed significant age-related trends in both sexes (*p* <0.05; *R*^2^ = 0.97–0.99). No consistent changes were observed across study years, apart from a slight decline among older women.

Elevated total cholesterol was common, affecting nearly two-thirds of participants overall. Prevalence was slightly higher in men (≈66%) than in women (≈64%; *p* <0.001; Table 4).

**Table 4:** Trends in elevated total cholesterol by year and sex.

Increased total cholesterol									
Year	Women				Men				*p*-value
	Yes		No		Yes		No		
	*n*	%	*n*	%	*n*	%	*n*	%	
2022*	73,606	63.53	42,262	36.47	44,710	66.01	23,022	33.99	<0.001
2023	223,802	64.36	123,941	35.64	149,226	66.38	75,582	33.62	<0.001
2024	164,238	64.43	90,679	35.57	117,737	66.86	58,363	33.14	<0.001

*Number of participants from July 1 to December 31, 2022.

**Figure 3:**
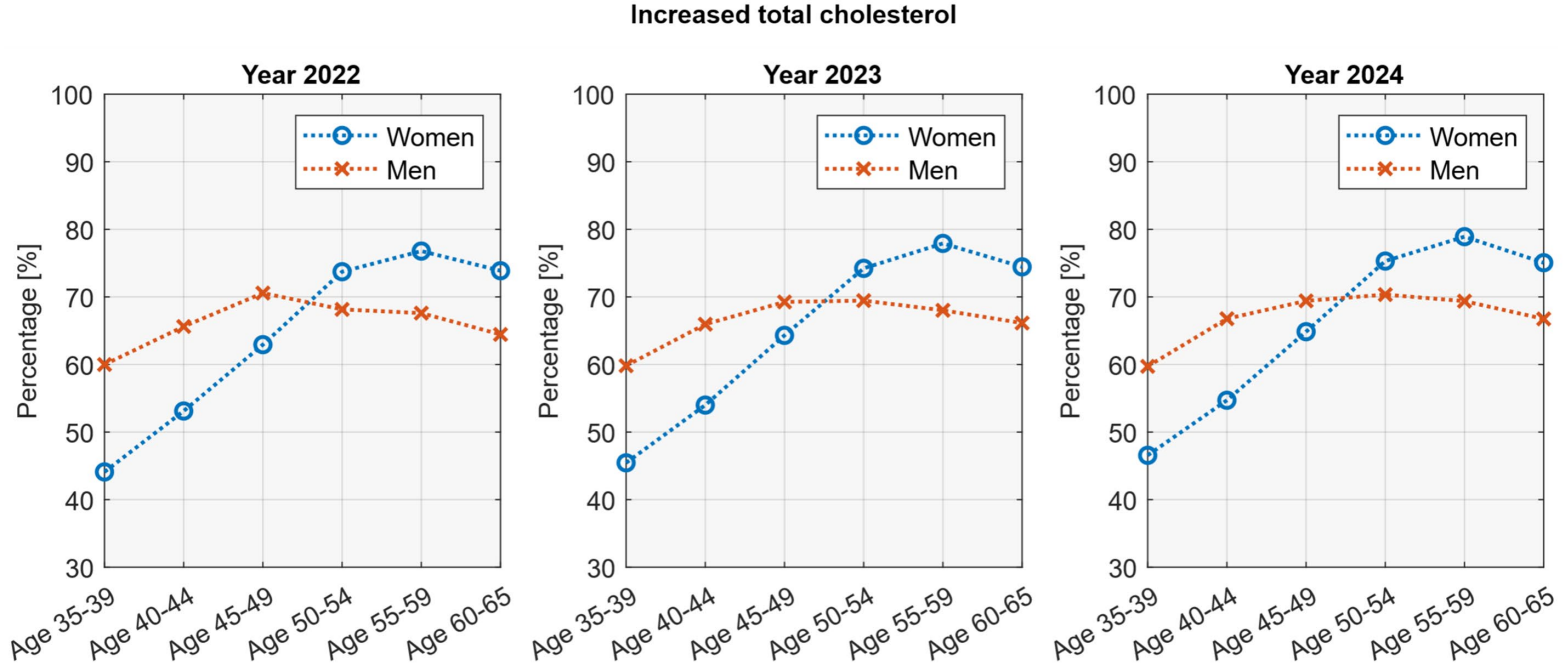
Age-stratified cholesterol rates by sex.

Age-stratified analyses showed distinct patterns between sexes (Figure 3). In women, elevated cholesterol increased steadily with age, peaking at 77–79% in those aged 55–59 years before declining slightly at 60–65 years (74–75%). In men, the peak occurred earlier, with 69–71% affected at ages 45–49, followed by a gradual decline in older groups. Polynomial regression models provided excellent fit for these non-linear relationships (*R*^2^ = 0.92–0.99) and confirmed the statistical significance of the age-related patterns (*p* <0.05 for both linear and quadratic terms). A temporal change was also observed in men, with cholesterol levels rising significantly between 2022 and 2024 (*p* = 0.047; *R*^2^ = 0.99). In women, a similar upward trend was seen but did not reach statistical significance (*p* = 0.29; *R*^2^ = 0.81).

Newly detected, previously undiagnosed diabetes was uncommon overall, reflecting screen-detected cases within a prevention-targeted cohort. Prevalence in men (2.9%) was more than twice that in women (1.2–1.3%; *p* <0.001; Table 5).

**Table 5:** Trends in the prevalence of newly diagnosed diabetes by year and age group in women.

Elevated glucose level indicating diabetic status (Women)													
Year	Age 35–39		Age 40–44		Age 45–49		Age 50–54		Age 55–59		Age 60–65		*p*-value
	*n*	%	*n*	%	*n*	%	*n*	%	*n*	%	*n*	%	
2022*	106	0.56	164	0.75	226	1.07	235	1.29	286	1.83	495	2.44	<0.001
2023	317	0.55	506	0.77	639	1.00	782	1.38	905	1.91	1,361	2.41	<0.001
2024	222	0.49	314	0.61	459	0.93	489	1.19	613	1.91	840	2.35	<0.001

*Number of participants from July 1 to December 31, 2022.

**Figure 4:**
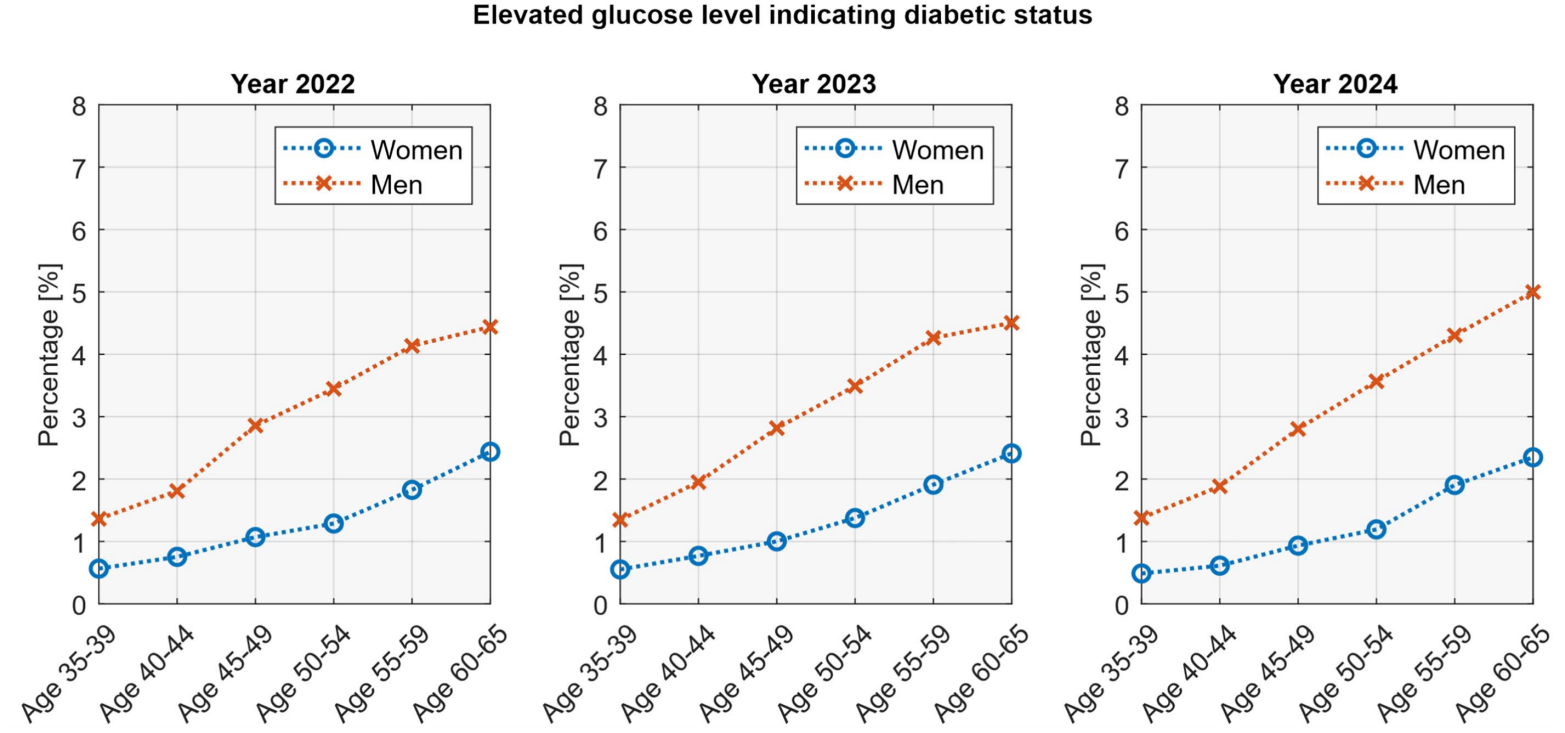
Prevalence of screen-detected diabetes by age group, sex, and year.

Elevated glucose increased with age in both sexes. In women, prevalence rose from around 0.5% at ages 35–39 to 2.4% at ages 60–65. In men, the increase was steeper, from about 1.3% at younger ages to nearly 5% at ages 60–65. Linear regression confirmed these age-related trends (*p* <0.05) with excellent model fit (*R*^2^ = 0.95–0.99). Notably, among older men (55–65 years), glucose levels showed a rising temporal change across the three study years (Figure 4).

### 3.3 Health behaviors

Smoking was more common in men than in women, affecting about 28% of men versus 19–20% of women (*p* <0.001; Table 6). Prevalence remained stable across study years, with no consistent temporal changes.

**Table 6:** Trends in smoking prevalence by year and sex.

Smoking									
Year	Women				Men				*p*-value
	Yes		No		Yes		No		
	*n*	%	*n*	%	*n*	%	*n*	%	
2022*	22,859	19.73	93,009	80.27	19,002	28.05	48,730	71.95	<0.001
2023	69,697	20.04	278,046	79.96	63,539	28.26	161,269	71.74	<0.001
2024	49,453	19.40	205,464	80.60	49,386	28.04	126,714	71.96	<0.001

*Number of participants from July 1 to December 31, 2022.

**Figure 5:**
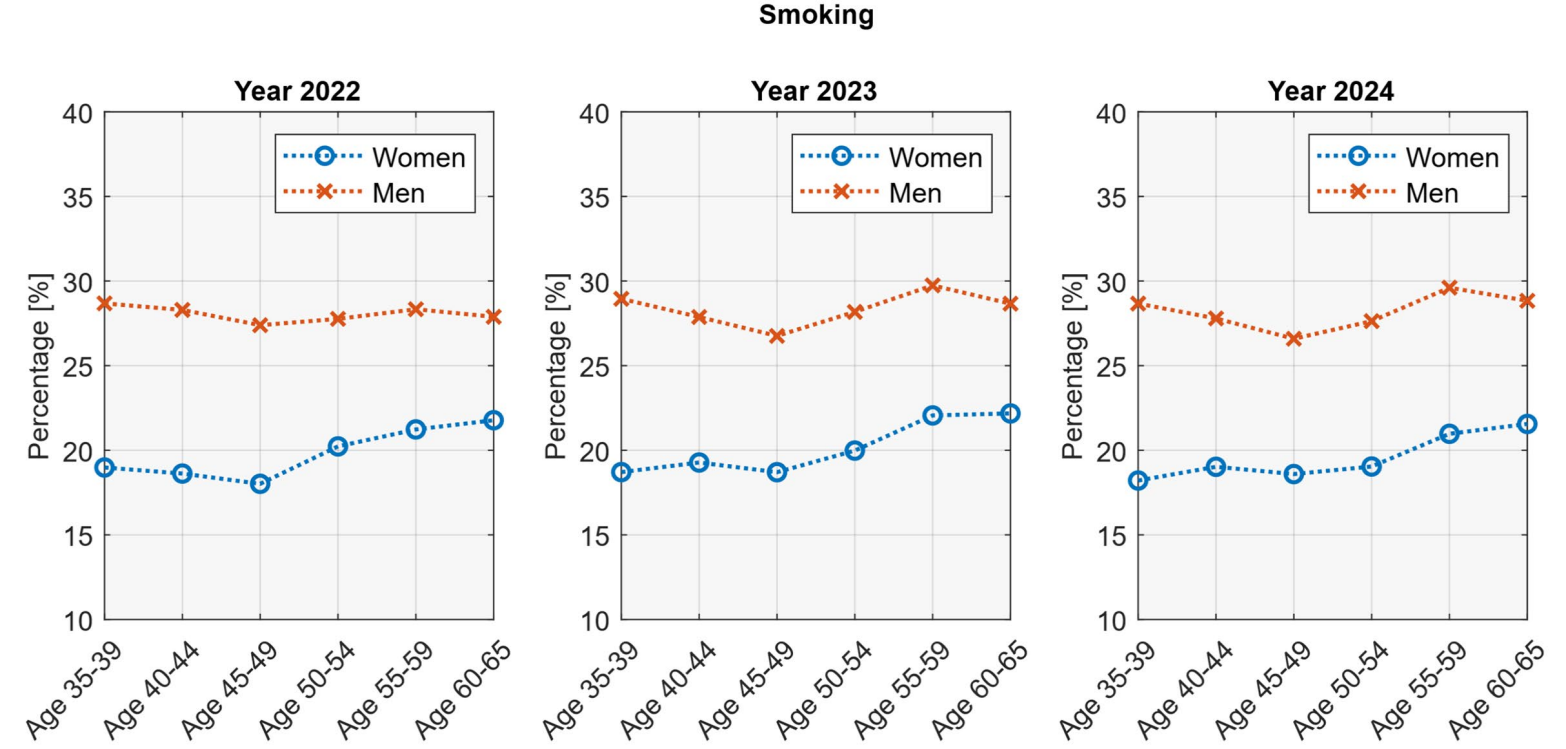
Age-stratified smoking rates by sex.

Age-related patterns in smoking differed by sex (Figure 5). In women, prevalence increased with age, rising after 45 years and reaching about 22% at ages 60–65 (*p* <0.05; *R*^2^ = 0.72–0.82). In men, smoking rates remained relatively stable across age groups, with no significant trend.

Low physical activity was common, affecting about half of participants overall. Rates were slightly higher in women (≈50–52%) than in men (≈49–51%; *p* <0.001; Table 7).

**Table 7:** Trends in low physical activity by year and sex.

Low physical activity									
Year	Women				Men				*p*-value
	Yes		No		Yes		No		
	*n*	%	*n*	%	*n*	%	*n*	%	
2022*	60,735	52.42	55,133	47.58	34,233	50.54	33,499	49.46	<0.001
2023	174,396	50.15	173,347	49.85	109,280	48.61	115,528	51.39	<0.001
2024	130,567	51.22	124,350	48.78	86,986	49.40	89,114	50.60	<0.001

*Number of participants from July 1 to December 31, 2022.

**Figure 6:**
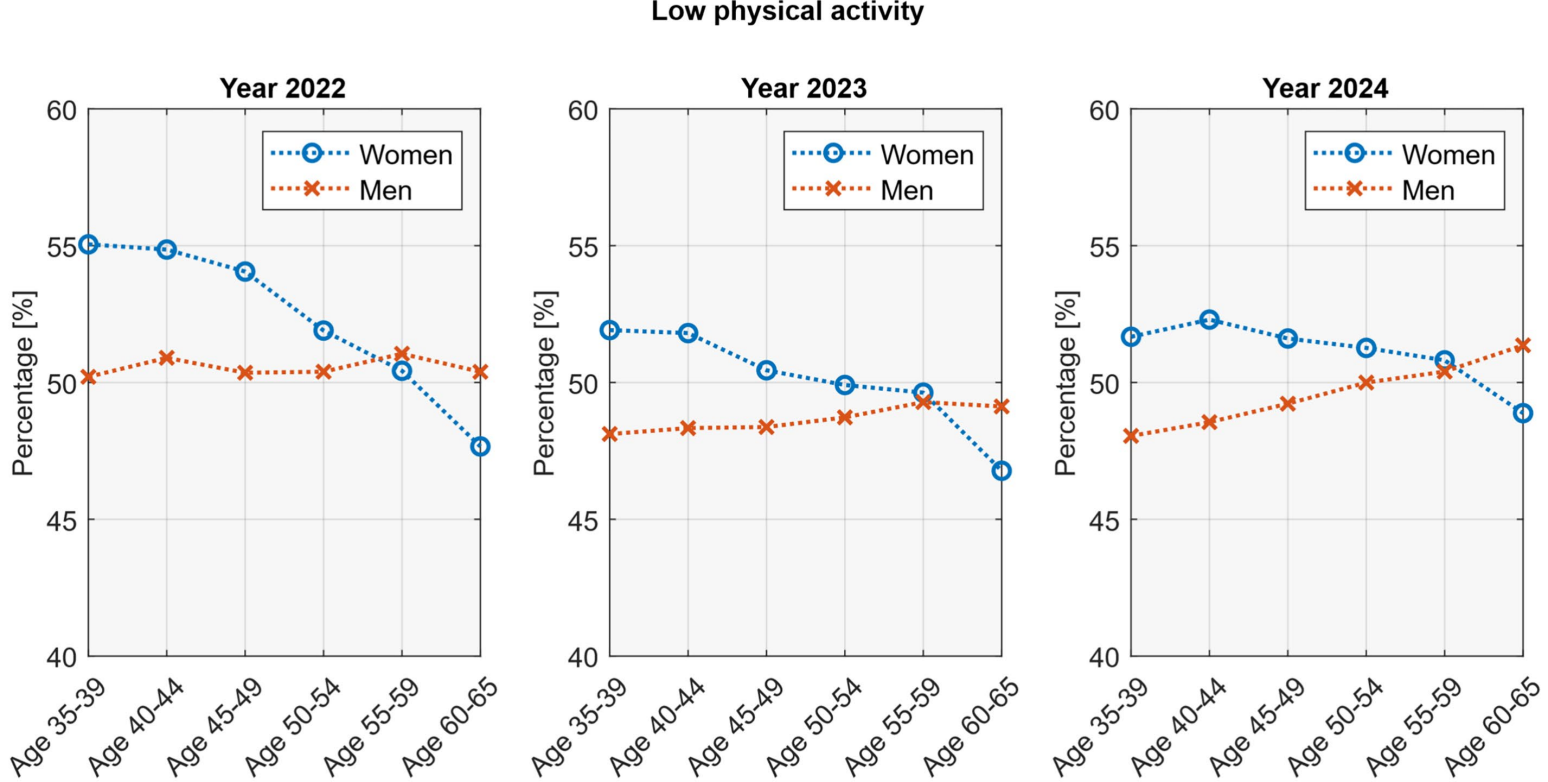
Age-stratified physical activity rates by sex.

Age-related patterns in physical activity differed by sex (Figure 6). In women, low activity declined with age, from about 52–55% at ages 35–39 to 47–49% at ages 60–65 (*p* <0.05; *R*^2^ = 0.71–0.93). In men, the trend was reversed, with prevalence increasing across age groups, particularly in 2023 and 2024 (*p* <0.05; *R*^2^ = 0.89–0.99).

## 4 Discussion

This large-scale analysis of cardiovascular risk factors among 1,187,168 participants in Poland’s CHUK prevention program from 2022 to 2024 provides important insights into population-level health patterns and their implications for targeted prevention. As in many European countries, women were significantly more likely than men to participate in preventive screening, consistent with findings from comparable initiatives across Europe (15–18). The prevalence of overweight and obesity was high, with clear sex-specific patterns. Among women aged 35–65 years, rates nearly doubled with age, whereas in men overweight remained stable and obesity declined after peaking in midlife. Hypertension prevalence appeared lower than expected, which may reflect selection bias in program participation.

Elevated cholesterol remained common, particularly among women, with no decline in older age groups, contrasting with studies where decreases are attributed to statin use. Undiagnosed diabetes also rose with age, with as many as one in 20 men aged 60–65 potentially affected. Smoking rates remained high across all age groups, with an upward trend among older women. In contrast, physical activity showed opposite age-related patterns by sex, increasing among women but declining in men. These differences may reflect sex-specific barriers and motivators, such as caregiving responsibilities among women and occupational activity patterns among men.

A key finding was the consistent and pronounced sex disparity across all cardiovascular risk factors, with men showing higher rates of hypertension, elevated glucose, overweight, obesity, and smoking than women. These results are consistent with prior epidemiological studies in Poland and across Europe, including findings from the EUROASPIRE V study and the European Health Examination Survey (EHES), and they emphasize the need for sex-specific prevention strategies (8, 19–21). The prevalence of hypertension in men was nearly twice that in women (≈30% vs. ≈16%), consistent with the 2025 PwC report estimating a national prevalence of 35% (22). Our estimates also exceeded those from the WOBASZ II study (43% in men vs. 33% in women) (23), which may reflect methodological differences or a shifting risk profile between sexes. This disparity has major implications for cardiovascular outcomes, as recognized in the National Program for Cardiovascular Diseases (NPChUK) 2022–2032 (24).

The sex gap in newly detected diabetes was even more pronounced, with men showing rates more than twice as high as women (2.9% vs. 1.2–1.3%). This aligns with the broader challenge of cardio-renal-metabolic syndrome, which recent public health initiatives have identified as a growing concern, with as many of adults at risk of related conditions (25, 26). The widening gap with age further suggests biological and behavioral differences in glucose metabolism that warrant tailored interventions.

Our analysis confirmed clear age-related patterns in cardiovascular risk factors, with most showing linear or polynomial associations with advancing age. The steady rise in hypertension prevalence in both sexes reflects vascular stiffening and endothelial dysfunction associated with ageing, consistent with established pathophysiological models. Particularly noteworthy was the non-linear trajectory of total cholesterol, which peaked earlier in men (≈45–49 years) than in women (≈55–59 years). This sex-specific pattern mirrors findings from other CEE populations (27), and highlights the region’s distinct cardiometabolic risk profile within Europe. In Poland, these results coincide with growing public health attention to lipid disorders. The designation of 2023 as the “Year of the Fight Against Hypercholesterolemia” (28) and the planned nationwide screening for familial hypercholesterolemia in children (“6-year-old health check”) in 2025 (22)–reflect alignment with EU goals to promote early detection and lifelong risk reduction.

### 4.1 Temporal changes and public health implications

The significant increase in total cholesterol among men over the three-year period (*p* = 0.047) is concerning and warrants further investigation. This trajectory runs counter to the NPChUK objectives of aligning Poland’s health indicators with EU averages (24). Recent initiatives such as the KOS-Lipid program integrated within the KOS-Zawał framework aim to tackle this issue through more effective treatment of hyperlipidemia (29). This upward trajectory also diverges from broader European efforts to reduce lipid-related cardiovascular risk through population-wide strategies and early therapeutic intervention (30).

Similarly, rising glucose levels in older adults, particularly men, suggest deteriorating glycemic control or gaps in diabetes management in these cohorts. This pattern is particularly concerning in light of data from the 2025 PwC report showing that diabetes affects approximately 9% of the Polish population (22). These patterns mirror trends across Europe, where cardiometabolic multimorbidity, particularly dysglycemia, is increasingly recognized as a cross-border challenge in ageing populations (31).

Persistently low physical activity levels in this cohort suggest that current public health campaigns are failing to engage large segments of the population. This finding aligns with the National Cardiology Network initiative, which highlights the importance of combining lifestyle interventions with medical treatment (32). Stagnation in behavioral risk factor modification, despite advances in pharmacotherapy, has also been observed elsewhere in Europe and underscores the urgent need for integrated prevention models (30). The divergent age-related trends in physical activity, declining with age in women but increasing in men, are rarely reported in previous studies and may reflect sex-specific barriers and motivators, such as caregiving responsibilities among women and occupational or social activity patterns among men.

### 4.2 Access to care and structural challenges

Our findings should be interpreted in the context of significant regional disparities in healthcare infrastructure and outcomes in Poland, as highlighted in recent reports (22). The 2025 PwC cardiovascular health report for Poland identified alarming variations in CVD mortality rates across different voivodeships, with some regions experiencing significantly higher rates than others (22). These regional variations may impact the detection and management of the risk factors identified in our study.

Additionally, the report identified challenges in coordination of cardiovascular care following screening and diagnosis, with ineffective pathways for patients outside the framework of the National Cardiology Network (32). This is particularly problematic for rural areas, where communities face additional barriers including limited awareness about diagnostic and therapeutic innovations, longer distances to hospitals, and compromised emergency care effectiveness (22). The recent initiative to establish Centers of Cardiological Excellence (Centra Doskonałości Kardiologicznej) within the National Cardiology Network, as outlined in the NPChUK, may help address these disparities by providing specialized centers focused on advanced cardiovascular care, research, and education (24).

These challenges are further exacerbated by long-term epidemiological and lifestyle trends specific to the Polish population. This epidemiological transition in Poland has been characterized by complex interactions between improving medical care and changing lifestyle patterns. While access to modern cardiovascular interventions has expanded, adverse changes in dietary habits, physical activity patterns, and stress levels have emerged as countervailing forces (33). According to recent data from 2024, Poland faces increasing demands for specialized cardiovascular care driven by high prevalence of risk factors such as hypercholesterolemia (affecting approximately 60% of adults), hypertension (35%), and hyperglycemia (9%), along with behavioral factors including smoking (25%), physical inactivity (50%), and increasing rates of overweight and obesity (31%) (22, 34).

The cardiovascular risk profile in Poland shows distinct features compared with other European nations. Historically, Poland has reported higher prevalence of hypertension and hypercholesterolemia than Western Europe (35) driven by dietary patterns high in saturated fats and sodium, lower levels of physical activity, and socioeconomic determinants shaping health behaviours (36, 37). This national pattern mirrors broader projections for Central and Eastern Europe, which is expected to reach the highest age-standardized cardiovascular mortality in Europe around 305 deaths per 100,000 population compared with 184 per 100,000 in Western Europe (38). Such forecasts underscore the urgency of addressing structural and systemic inequities and reinforce the importance of aligning national strategies with broader pan-European prevention goals.

### 4.3 Data limitations and future directions

While our study provides valuable insights, the cross-sectional nature of the data precludes causal inference, and potential selection bias should be considered as CHUK program participants may not be representative of the general Polish population. Smoking status and physical activity were assessed by self-report, which may have resulted in underestimation of smoking prevalence and overestimation of physical activity levels. Additionally, the 2022 data covering only July–December limits direct comparability with the full-year data from 2023 and 2024. These limitations underscore the need for improved data collection and analysis systems, as highlighted in the 2025 PwC report (22). The report emphasized suboptimal use of data and analytics for decision-making in Poland’s cardiovascular health management, noting that despite improvements in the availability of patient data, significant gaps remain in integration and standardization across various data sources (22). Such systemic data fragmentation is not unique to Poland; it reflects broader challenges across European health systems striving to modernize their digital health infrastructures while ensuring interoperability, equity, and data security. The proposed electronic Cardiology Care Card (elektroniczna Karta Opieki Kardiologicznej eKOK) in the draft of National Cardiology Network legislation represents a positive step toward creating a universal and standardized source of information for patients with CVD^32^. Its successful implementation could serve as a model for integrated cardiovascular care pathways, consistent with the European Commission’s goal of fostering cross-border data continuity and supporting digital public health ecosystems. Furthermore, dietary intake was not assessed, precluding adjustment for nutritional determinants of cardiovascular risk. Only baseline data were available, with no possibility of longitudinal follow-up or registry linkage, which restricted the scope of analyses to risk factor prevalence. The relatively short observation period limits the interpretation of temporal changes as long-term trends. Finally, the use of different certified devices across healthcare settings may have introduced minor measurement variability; however, all equipment met mandatory medical and regulatory standards.

### 4.4 Implications for policy and practice

Our findings support several key recommendations from recent cardiovascular health initiatives in Poland. First, they underscore the importance of adopting systematic and long-term strategies for cardiovascular screening and prevention, backed by sustained funding and coordinated action at the national level (22). Second, the National Cardiology Network offers a platform to harmonize patient care pathways across regions, particularly for individuals with newly diagnosed or chronic cardiovascular conditions (32).

Third, there is a pressing need to establish a centralized institution responsible for managing cardiovascular health data to ensure consistency and quality in data collection and analysis nationwide (22). Regular review and revision of reimbursement policies will also be essential to secure equitable access to cardiovascular therapies for all patient groups, regardless of socioeconomic or regional disparities (39). Finally, the planned rollout of dedicated health education in Polish schools in September 2025-alongside the Senate of the Republic of Poland’s declaration of 2025 as the Year of Health Education and Prevention-represents a critical opportunity to address modifiable cardiovascular risk factors through early education and sustained prevention efforts (40).

Collectively, these insights may inform not only national efforts but also contribute to Europe-wide strategies aimed at reducing preventable cardiovascular mortality through harmonized, equity-driven prevention.

## 5 Conclusion

Our findings reveal a persistent and deeply stratified cardiovascular risk profile in Poland, marked by substantial sex disparities and progressive age-related deterioration in key health indicators. The particularly high burden among middle-aged men suggests that current prevention frameworks may be failing to engage the groups at greatest risk. The observed temporal increases in cholesterol and glucose levels, despite national efforts, underscore the need for more responsive and adaptive prevention models that go beyond routine screening. Risk is not static and neither should be the public health response. To reduce the long-term burden of cardiovascular disease, future efforts must prioritize implementation science: transforming population data into tailored interventions that account for social, behavioral, and structural determinants of health. Integrating such insights into the design of national programs could help bridge the persistent gap between identification of risk and its effective management.

## Data Availability

The raw data supporting the conclusions of this article will be made available by the authors, without undue reservation.
